# Image Semantic Recognition and Segmentation Algorithm of Colorimetric Sensor Array Based on Deep Convolutional Neural Network

**DOI:** 10.1155/2022/2439371

**Published:** 2022-09-30

**Authors:** Jingjing Tang, Li Wang, Jing Huang, Aiye Shi, Lizhong Xu

**Affiliations:** ^1^College of Computer and Information, Hohai University, Nanjing 211100, Jiangsu, China; ^2^Business School, Hohai University, Nanjing 211100, Jiangsu, China

## Abstract

Semantic feature recognition in colour images is required for identifying uneven patterns in object detection and classification. The semantic features are identified by segmenting the colorimetric sensor array features through machine learning paradigms. Semantic segmentation is a method for identifying distinct elements in an image. This can be considered a task involving image classification at the pixel level. This article introduces a semantic feature-dependent array segmentation method (SFASM) to improve recognition accuracy due to irregular semantics. The proposed method incorporates a deep convolutional neural network for detecting the semantic and un-semantic features based on sensor array representations. The colour distributions per array are identified for horizontal and vertical semantics analysis. In this analysis, deep learning classifies the uneven patterns based on colour distribution, i.e. the consecutive and scattered colour distribution pixels in an array are correlated for their similarity. This similarity identification is maximized through max-pooling and recurrent iterations, preventing detection errors. The proposed method classifies the semantic features for further correlation sections, improving the accuracy. The proposed method's performance is thus validated using the metrics precision, analysis time and *F*1-Score.

## 1. Introduction

Image semantic is a process that segments the pixels in an image within its region, and semantic values are given to each pixel with a specific label. Image semantics provide necessary information related to gasps and clusters in an image. In image segmentation, a strong approach known as clustering has been developed. An image data set can be partitioned into a number of distinct groups or clusters using a cluster analysis. Image semantic recognition is a complicated and challenging task in an image processing system [1]. Image semantic recognition provides relevant details for the analysis process that reduces the latency rate in the searching process. Image semantic recognition is most widely used in video and image analysis [2]. Sparse segmentation is used in the recognition process that finds the exact clusters and pixels of images. The sparse segmentation method summarizes the characteristics of images and provides the necessary set of data for the semantic recognition process [3]. Sparse segmentation increases the recognition process's accuracy rate, reducing the error rate in image semantics. Semantic segmentation makes it possible to distinguish between several types of things. The phrase “image segmentation” describes the act of breaking up a single picture into many smaller parts. Each image's pixel is assigned to a certain object type using this method. In image segmentation, semantic and instance segmentation are the two main approaches.

All objects of the same kind are labelled with a single class label in semantic segmentation, whereas related objects are labelled with distinct labels in instance segmentation. The long short-term memory (LSTM) algorithm is also used in the image semantic recognition process. LSTM leverage the characteristic and pixels of an image that improves the performance and feasibility of the system [4, 5].

A metric colour sensor detects particular arrays by identifying the colour-changing pixels in an image. Colorimetric sensors detect particular changes which occurred due to colour variation. Colorimetric sensors are optical sensors that also change some stimuli in an image [6]. The colorimetric sensor array is used in the image segmentation process that provides an appropriate set of data for the segmentation process. Colorimetric sensor arrays are used to identify the complex components presented in an image [7]. Various methods and techniques are used for the segmentation process. The fuzzy C-means clustering (FCM) algorithm is mostly used for the image segmentation process that enhances the system's feasibility [8]. Sensor arrays are used here that contain details about clusters and pixels of an image. FCM reduces the error rate, improving the computation process's accuracy rate. FCM classifies the pixel's value by identifying the labels. Principle component analysis (PCA) is used in the segmentation process that uses a sensor array. PCA increases the accuracy rate in the segmentation process, improving an image processing system [9, 10].

The image semantic recognition process mostly uses machine learning (ML) and a deep learning approach to find out the actual segment of an image. ML techniques are widely used for the detection and recognition process to improve the accuracy rate in the detection process [11].

An artificial intelligence (AI) technique known as machine learning (ML) enables software programmes to improve their ability to anticipate outcomes without having to be programmed. Machine learning algorithms use past data as input to forecast new output values. The convolutional neural network (CNN) algorithm is used in the image semantic recognition process. The CNN approach trains the dataset with a previously recorded data set and finds the difference in the recognition process [12]. The feature extraction process is used in CNN to extract the important features from an image. Various features and details are extracted from the image and provide necessary information for the classification process [13]. The classification process classifies the optimal semantic values and classes from the image. Classified classes are used here to form a separate image by using CNN. CNN approach improves the accuracy rate in the image semantic recognition process [10, 14]. CNN also increases the recognition process's effectiveness and efficiency rate, enhancing the system's feasibility. A deep convolutional neural network (DCNN) is also used in the image semantic recognition process. The segmentation method is used in DCNN to find out the actual semantic values of a pixel that provide appropriate details for the recognition process [12, 15].

The main contribution of this paper is semantic segmentation, which is a method for distinguishing between several things in an image. Pixel-level image classification can be viewed as a task. Semantic image segmentation aims to classify each pixel of an image with a matching class of what is being shown. Dense prediction is the term used to describe the process of making predictions for every pixel in an image.

The rest of the paper is as follows: Section 2 for a literature survey of the existing method, Section 3 proposed method for SFASM to be discussed, Section 4 for experimental analysis and Section 5 conclusion.

## 2. Related Works

Lau et al. [16] proposed a generative single-frame restoration algorithm for the face recognition process. The proposed algorithm is mainly used to reduce the deformation and blurriness that occur due to an image's turbulence. Fusion features are used here to find out the important features that are present in an image. The fusion feature provides the necessary set of data for the face recognition process. The proposed algorithm achieves high effectiveness, improving the system's performance and feasibility.

Rao et al. [17] introduced a bidirectional guided attention network (BGA-Net) for the semantic detection process in remote sensing images. The semantic segmentation module (SSM) is used for the segmentation process that estimates an image's maps and important features. The proposed BGA-Net model first trains the dataset needed for the recognition process and provides optimal details for the semantic detection process. A unified backbone module (UBM) is used here to maximize the performance and efficiency of an image processing system.

Cao et al. [18] proposed a head-level ensemble network (HENet) for the remote sensing image semantic segmentation. The semantic segmentation process uses very high-resolution (VHR) images to find out important features. The proposed HENet model reduces the complexity rate of an image processing system that maximizes its feasibility. The ensemble learning approach is used here to tackle the computation process problems that reduce the feature extraction latency rate.

Liu et al. [19] introduced a lightweight semantic segmentation network for unmanned aerial network (UAV) remote sensing images. The convolutional neural network (CNN) algorithm is used here to reduce the number of parameters in the semantic segmentation process. Attention models collect global semantic information. The lightweight model is mainly used here to predict an image's pixel and find out each pixel's quality. The proposed method achieves a high effectiveness and efficiency rate of the system.

Lin et al. [20] proposed a switchable context network (SCN) for semantic segmentation. The proposed method is used for RGB-D images to find semantic information from an image. SCN analyses an image's information and important regions and then provides an appropriate data set for the semantic segmentation process. SCN reduces the optimization problems that are available in an image processing system. Identifying image regions is a complicated task to perform in the semantic segmentation process. The proposed SCN model increases the feasibility and performance of the system.

Rose et al. [21] introduced a convolutional neural network (CNN) for an automated semantic segmentation system. CNN is mostly used for the prediction process that predicts the important features of an image and provides the necessary data for the segmentation process. Counting black pixels is an important task to perform in the semantic segmentation process. CNN increases the accuracy rate in the prediction process, enhancing the system's effectiveness. The proposed model improves the system's reliability, feasibility and efficiency.

Li et al. [22] proposed a deep semantic segmentation network (DSSN) for the remote sensing image semantic segmentation process. A collaboratively boosting framework (CBF) is implemented here to combine the DSSN model. The classification method is used here to find out the important features available in an image. Ontology reasoning modules are used here to find the image structures that provide the necessary information for the semantic segmentation process. The proposed model reduces the optimization problem rate, enhancing the detection and prediction accuracy rate.

Guo et al. [23] introduced a multilevel semantic adaption (MSA) for the few-shot segmentation process. MSA first identifies the semantic features that are available in the cardiac image. A hierarchical attention metric is used here to determine an image's frame-level features. The proposed multilevel model finds out an image's pixel level, regions and features. MSA addresses both weight and domain adaption of an image that provides an optimal set of data for the semantic segmentation process. Experimental results show that the proposed MSA model improves the feasibility and performance of the system.

He et al. [24] proposed semantic object segmentation and depth estimation network (SOSD-Net) for monocular images. The object assumption technique is used here to find the important features available in an image. The proposed method improves the accuracy rate in the monocular depth estimation process. The maximization algorithm is used here to increase the efficiency level of the semantic segmentation process. The proposed SOSD-Net model maximizes the overall performance rate of the system.

Alam et al. [25] introduced a convolutional neural network (CNN) algorithm for remote sensing image semantic segmentation. CNN algorithms are a deep learning approach that improves the effectiveness level of the remote sensing process. Encoder and Decoder algorithms are also used here to find out the semantic features of an image. Differentiate among objects are also identified by the modules used in the CNN algorithm. The proposed CNN model provides a better segmentation process that enhances the system's reliability.

Zhang et al. [26] proposed a deep-recursive residual network for the image semantic segmentation process. The recurrent convolutional neural network (RCNN) approach is used here to find out the important semantic features of an image. The feature extraction process provides the necessary data set for the RCNN approach. The proposed method improves the semantic segmentation process's accuracy rate, which increases the system's performance rate. The proposed method also reduces the parameters of the computation process.

Zhang et al. [27] introduced a convolutional neural network (CNN)-based image semantic segmentation process. The proposed CNN model mostly identifies an image's RGB colour, providing an optimal data set for the segmentation process. CNN finds the regions and features of semantic images that reduce the computation process's latency rate. Semantic classification classifies the important features and produces a feasible data set for further process. The proposed CNN model improves the overall performance rate of the system.

Yang et al. [28] proposed a stack space auto-encoding (SSAE)-based model for the image semantic process. SSAE finds out the important semantic information of an image and provides data for the segmentation process. A convolutional neural network (CNN) is also used here to identify the low-level features presented in an image. The proposed model increases the accuracy rate in the segmentation process, improving the system's overall effectiveness.

Zhu et al. [29] introduced an improved position attention model for semantic image segmentation. The feature extraction process extracts the necessary set of features from an image. A spatial pooling pyramid (SPP) is used here to analyse the features extracted from the feature extraction process. The position attention model is integrated here to remove the trivial information presented in an image. The proposed model improves the accuracy rate in identifying semantic information from an image, achieving better performance. Compared with the existing method, the proposed method improves accuracy, high precision, *F*1-Score, less error ratio and less analysis time.

## 3. Proposed Semantic Feature-dependent Array Segmentation Method

**Figure 1:**
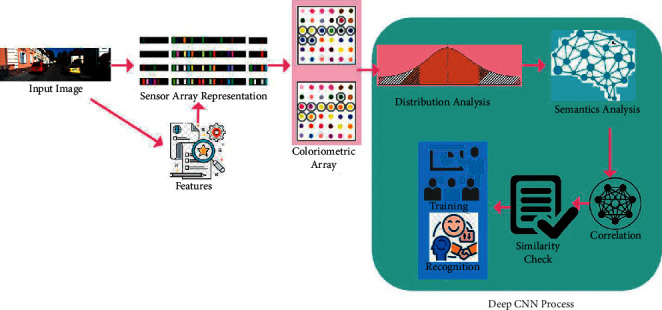
Proposed method.

The proposed SFASM method is designed to improve the semantic feature recognition accuracy due to identifying uneven patterns in object detection and classification based on colour images. Input image recognition and segmentation refer to sensor array representation and colorimetric array based on a semantic feature, such as colour, texture, greyscale and shape. These features appear similar in the same and different regions. The semantic features are identified based on colorimetric sensor array feature inputs. The semantic identification between sensor array representation and a colorimetric array is required from the features. An optical sensor that changes colour in response to external stimuli is a colorimetric sensor. Any change in the environment is a stimulus. Sensors sense and react to a certain form of physical input from their surroundings. Light, heat, motion, wetness, pressure and a slew of other environmental phenomena can all serve as inputs. An array's distribution of coloured pixels is observed at different time intervals through a machine learning paradigm that detects semantic and un-semantic features based on sensor array representation. In particular, the analysis can automatically recognize and segment objects existing in the input image. The conventional semantic image recognition and segmentation analysis are based on spectral clustering. According to the different image pixels, the input image is divided into two categories based on semantic analysis.  Figure 1 presents the proposed method.

The feature-based on semantic analysis depends on colour distributions per array on a deep convolutional neural network. The main role of this method is to reduce detection errors in a colorimetric sensor array. The challenging factor in this proposed work is semantic image recognition and segmentation analysis based on the sensor array representation with the new input image instances. The semantic image is stored as a colorimetric array from the previous image recognition and segmentation based on sensor array representation. Image segmentation is a technique used to reduce the complexity of a digital image so that it can be processed or analysed more straightforwardly in the future. Segmentation is the process of labelling individual pixels. The sensor array and colorimetric array representation require semantic features based on colour distribution analysis performed for horizontal and vertical semantic analysis. The semantic feature analysis of input images based on colour distribution pixels in an array is accessed for performing correlation depending on their similarity check. It classifies the uneven patterns based on colour distribution through a deep convolutional neural network. The consecutive and scattered colour distribution pixels are correlated for their similarity check. The similarity identification reduces the training and increases the recognition through max-pooling and recurrent iterations. The main role of this image recognition and segmentation modelling is to increase similarity identification and colour distribution of the current input image based on sensor array representation. In the context of colour image segmentation, it is assumed that homogenous hues correlate to discrete clusters and meaningful objects in the image. A group of pixels with similar colour qualities is defined as a cluster. The new image is based on the classification of semantic features for further correlation analysis through a deep convolutional neural network. With the consecutive improvement of the deep convolutional neural network, the image semantic recognition and segmentation have been further developed, the semantic features have been extracted accurately and immediately, and the recognition outputs in more accurate.

### 3.1. Conventional Image Semantic Recognition and Segmentation

The proposed machine learning paradigm for input semantic image recognition and segmentation is based on the semantic features identified by segmenting the colorimetric sensor array features. Compared with the conventional minimization of image recognition analysis, the normalized image semantic recognition not only satisfies the minimum object detection. Semantic segmentation provides a pixel-level classification of an image. At the same time, object detection classifies the patches of an image into distinct object classes and builds a bounding box around that object. The classification between uneven patterns also satisfies the maximum object detection and classification. This is defined as follows:

(1a) $$\begin{array}{c} S_{\text{image}\left(a,b\right)}=\frac{\text{Image}\left(a,b\right)}{{\text{assoc}}_{\text{Image}\left(a,f\right)}}+\frac{\text{Image}\left(a,b\right)}{{\text{assoc}}_{\text{Image}\left(a,f\right)}}, \end{array}$$

where the semantic features are identified as below:

(1b) $$\begin{array}{c} \text{Image}\left(a,b\right)=\sum_{x\in a,y\in b}{\text{Color}}_{s\left[\text{array}\right]}\left(x,y\right). \end{array}$$

**Figure 2:**
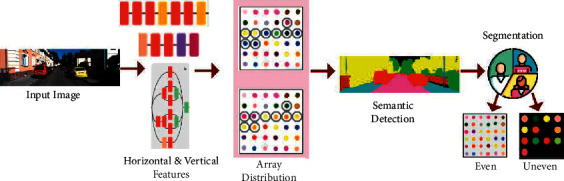
Semantic segmentation process illustration.

As per the equations (1a) and (1b), where the variables *a* and *b* are the two disjoint sets in the input semantic image of *S*_image(*a*, *b*)_, and *f* are used to represent the semantic features. Where *a* ∪ *b*=*f*, *a*∩*b*=∅, Color_*s*[array]_(*x*, *y*) represents the sum of colorimetric sensor array between *x* and *T* nodes assoc_Image(*a*, *f*)_=∑_*x*∈*a*,*y*∈*b*_Color_*s*[array]_(*x*, *y*). In this equation, where *x* represents all nodes in *a*, similarly, *y* represents all nodes in *y* and *T* represents all nodes in the input semantic image. However, in practical applications, the image semantic recognition and segmentation algorithm can only perform the identification of uneven patterns in the input image once per execution. An image segmentation approach reduces the complexity of an image for further processing or analysis by dividing it into smaller groupings called Image segments. Segmentation is the process of assigning labels to individual pixels. A label is provided to all image parts or pixels in the same category. Therefore, it is identified when the input image contains uneven patterns; this algorithm must be executed successively many times, generating a solution in inaccurate segmentation output. In Figure 2, the semantic segmentation process is illustrated.

The input image is extracted for its horizontal and vertical features using (*a*, *b*). From the extraction, array distribution using colorimetric representation is performed. The horizontal to vertical distribution and vice versa identify even and uneven input segmentations (Refer to Figure 2). The proposed semantic image recognition and segmentation method used a machine learning paradigm to obtain a colorimetric sensor array. Then semantic features are used to extract the colour distribution pixels of each sensor array, eventually uses *S*_image(*a*, *b*)_ to get image recognition and segmentation outputs. It not only used the colour distribution pixel information of the pursued input image and also considered the semantic analysis based on boundary information, which guaranteed the image semantic recognition and segmentation effect. This proposed image semantic recognition and segmentation algorithm is based on machine learning and secondary segmenting of the colorimetric sensor array. This algorithm combined semantic and un-semantic features based on sensor array representations. First, it uses object detection to perform the distribution analysis, which would guide horizontal and vertical semantic analysis based on the semantic features. The algorithm was robust and had good performance on uneven patterned images. A weakly supervised colour distribution per array is identified for coloured pixel images. The image semantic recognition and segmentation method use colour distribution analysis and is given as

(2) $$\begin{array}{c} \max\sum_{x\in a,y\in b}D_{CA}{\left(x,y\right)}^{T}. \end{array}$$

Such that

(3) $$\begin{array}{c} \sum_{T\in D_{CA}}SF=\prod_{T=ab}SF-\left[\frac{P_{s}}{\sum \left(SF_{A}+U_{S}F_{A}\right)}\right]. \end{array}$$

As per the equations (2) and (3), the variables *D*_*CA*_, *SF*, *SF*_*A*_ and *U*_*S*_*F*_*A*_ are used to denote colour distribution analysis, semantic feature identification and semantic and un-semantic feature analysis. It is based on colorimetric sensor array representation through a deep convolutional neural network. The condition *D*_*CA*_(*x*, *y*) is used to denote the semantic image availability based on sensor array analysis between a colour distribution and semantic analysis at a different time interval. The maximum semantic feature of *T*=1 achieves high recognition accuracy *R*_*α*_ for the horizontal and vertical semantic image analysis based on colour distribution. Instead, the colour distribution and semantic feature analysis are not stable due to *T* ∈ [0,1] be the varying constraint. Instead, *T*=1 is not ensuring the object detection and classification process at any interval resulting in object detection error in the input image. This problem is called detection error in semantic feature recognition of colorimetric sensor array based on a DCNN. These uneven identifying patterns in object detection and classification rely on the input image semantic feature analysis. The colour distribution analysis is jointly used in this scheme to maximize recognition accuracy through semantic analysis. A colour histogram visually depicts how an image's colours are distributed. A colour histogram depicts the number of pixels in an image's colour space, the set of all possible colours that have a colour in each of a given list of colour ranges.

### 3.2. Semantic Analysis Based on Colour Distribution Pixel

**Figure 3:**
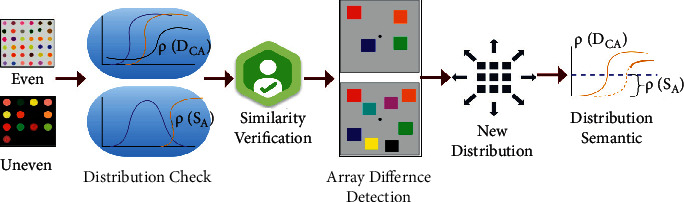
Distribution process illustration.

In semantic analysis based on colour distribution, the input images' horizontal and vertical semantic analysis classifies the uneven patterns based on colorimetric sensor array representation based on colour distribution. Initially, this proposed method identifies the new colour distribution per array based on vertical and horizontal lines in the semantic feature analysis. The colour distribution pixels in an array are correlated for similarity analysis based on semantic identification. The distribution process is illustrated in Figure 3.

The even and uneven segmented outputs are verified for this array distribution ∀*ρ*(*D*_*CA*_) and *ρ*(*S*_*A*_) (Refer to Figure 3). This is independently performed until a maximum *S*_*v*_ is achieved. Extracting the difference in colorimetric array distribution, a common distribution assimilating *S*_*A*_ and *D*_*CA* _ is performed. The assimilated distribution is verified for further array assignments based on the semantics detected. Therefore, the similarity verification is processed through horizontal and vertical semantic analysis relying on the sensor array representations as in equation (1a). The probability of distribution and semantic analysis depends on the input image in *D*_*CA*_ without detection errors; therefore, *ρ*(*D*_*CA*_, *S*_*A*_) is discussed below

(4) $$\begin{array}{c} \rho \left(D_{CA},S_{A}\right)=\frac{\prod_{T=D_{CA}}SF_{T}}{\prod_{T=ab}D_{\text{CAT}}+\sum \left(SF_{A},U_{S}F_{A}\right)}. \end{array}$$

In equation (4), the colour distribution and semantic analysis are based on the input image features in sensor array representation at different time intervals. The actual uneven patterns identification of the different image semantic features based on colour distribution pixels *C*_*P*_ in an array are compared with the semantic analysis, respectively. In particular, the object detection and correlation based on semantic image recognition of 1 − [*D*_*CA*_/∑(*SF*_*A*_, *U*_*S*_*F*_*A*_)] is estimated using *C*_*r*_. Semantic feature analysis computes the correlation between a colour distribution and semantic analysis. This correlation instance based on consecutive and scattered colour distribution identifies the semantic features. This semantic feature is classified as semantic and un-semantic analysis based on colorimetric sensor array through deep convolutional neural network learning is estimated as

(5) $$\begin{array}{c} C_{P}\forall T\in D_{CA}\cup^{}SF=\left[\left[\left(1-C_{r}\right)\frac{C_{P}}{U_{S}F_{A}}\right]-\left[T*C_{P}-\left(H-V\right)\right]\right],\;T\in SF. \end{array}$$

In equation (5), the variables *H* and *V* denote the horizontal and vertical semantic analysis based on the colour distribution pixels and correlation *SF* compared with other images and perform either *H* or *V* based on the sensor array. The object detection and classification of an input image at the initial and final level are processed to maximizing *C*_*r*_. Based on the condition *C*_*P*_∀ *T* ∈ *H*∪*V* exceeds, and then the semantic feature recognition and segmentation analysis are performed. These functions are dependent on correlation with the colour distribution pixels and uneven pattern identification. The consecutive and scattered colour distribution is analysed based on the input image handles the array segmentation method and coloured pixels for an array of *C*_*P*_ as (*T*, *SF*, *ρ*(*D*_*CA*_, *S*_*A*_)). This array is correlated for the similarity check or modifies the semantic features in *D*_*CA*_ for all the *x* and *y* nodes. This output is considered for correlation for new images, and features can be changed based on even patterns. In this manuscript, the outputs are used for recognition and training, and it relies on *D*_*CA*_ and *T* for the correlation instance in the above-discussed equation. Let  Recog and  Training denote the recognition and training instance in similarity verification based on the sensor array at the initial level. It refers to the new semantic feature analysis and colour distribution pixel changes for the uneven pattern images relying on different semantic features and pixels. Semantic features include everything that can be seen in the image, such as form, colour, type, etc. Problems with text visibility need a comprehension of the semantic characteristic, just as they would in a picture with a large number of people. To bridge the semantic gap between low-level visual elements and high-level ideas that capture the transmitted meaning, image analysis at a semantic level results in the automated extraction of picture descriptions according to human perception. Therefore, the similarity verification is based on horizontal and vertical correlation process through max-pooling and recurrent iterations performed for *S*_*V*_ is given as

(6) $$\begin{array}{c} S_{V}=\text{Recog}\left(H*V-\frac{D_{CA}}{SF}\right)+\text{Train}\left(\frac{H}{V}\right)\forall T\in C_{r}, \end{array}$$

where

(7) $$\begin{array}{c} \left.\begin{array}{c} \text{Recog}=\sum_{T\in D_{CA}}D_{CA}=ab\sum_{T\in SF}\frac{D_{CA}}{S_{A}}-\left[H-V\right]\\ \text{and}\\ \text{Training}=\sum_{T\in SF}D_{CA}^{T}-\left(1-C_{r}\right)=\sum_{T\in D_{CA}}\left(H-V\right) \end{array}\right\}. \end{array}$$

**Figure 4:**
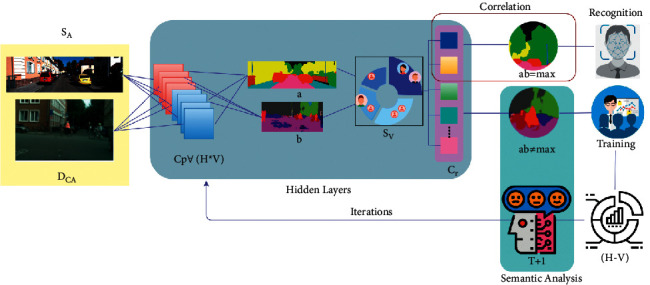
Learning process for recognition and correlation.

The above equations (6) and (7), *S*_*V*_ is computed as a circumstance of colour distribution pixels with correlation to find the accurate recognition. Therefore, this semantic feature recognition is responsible for horizontal and vertical semantic features. It performs correlation based on a deep convolutional neural network depending on image semantics. The segmentation process strengthens the recognition with fewer detection errors and analysis time for their similarity check. The recognition and training are designed for the correlation section based on semantic feature analysis. The fluctuating condition of training and recognition based on semantic features is correlated and then checked for similarity with the previous segmented image based on the colour distribution in sensor array processing. Based on this consecutive manner, the conventional semantic feature recognition in image processing provides high accuracy for colour distribution. The similarity verification is analysed with training and recognition of image semantics through a neural network with the help of a colorimetric sensor array. The learning process for recognition and correlation is illustrated in Figure 4.

The learning method intakes *S*_*A*_ and *D*_*CA* _ inputs for various *C*_*P*_∀ *H* and *V* conjointly. This intake is classified as (a, b) as in equations (1a) and (1b). From this classification, *S*_*v*_ is performed independently ∀*C*_*r*_ and the conditions *ab*=max and *ab* ≠ max are identified. The maximum condition satisfaction recognizes the object, whereas the failing condition requires (*T*+1) iteration. These two processes are recurrently performed until (*H* − *V*)=0 is achieved (Refer to Figure 4). The semantic feature dependent on the array and colour distribution is analysed, and similarity verification is performed based on the correlation. This correlation of colour distribution pixels as per array is either of *H* (or) *V*, in both instances, if *D*_*CA*_=0, then the semantic feature is identified by segmenting the colorimetric sensor array as *D*_*CA*_=*SF*=*C*_*r*_ is the recognition maximizing condition, and if *D*_*CA*_=1, and then *SF*=*C*_*r*_ − *D*_*CA*_ and *SF*=*C*_*r*_. Therefore, the occurrence of *C*_*r*_=*SF* is a reliable output for image semantic recognition. The depreciation of error detection in all the colour distribution and semantic analysis with the sensor array representation is derived in equations (1a) and (1b). The image recognition is high; less training is provided for further analysis. In contrast, the recognition decreases, then training increases for the input image and finally correlated through the deep neural network. Therefore, the semantic feature is identified as per the colorimetric sensor array. In any instance of performing correlation, if the condition is analysed *H* < *V*, then the image satisfies maximum object detection and classification, for instance, which again results in segmentation. The input image based on semantic features in both the condition of Recog and  Training is estimated conventionally based on correlation instance to ensure *H* > *V*. Based on the segmentation method, the computation of semantic feature recognition analysis for all the image semantics differentiates the semantic and un-semantic features through deep CNN for semantic analysis based on colour distribution. The image semantic recognition and segmentation algorithm is available for performing the recognition and training for images. Therefore, the input image semantics are based on colorimetric pixels of an array for the next image. If the semantic image recognition increases, the consecutive and scattered colour distribution is high in this condition. At the same time, semantic image training increases the consecutive and scattered colour distribution less than the other feature in object detection. Therefore, the minimum training and error detection in image semantics are achieved. Therefore, the colour distribution and semantic feature analysis are consecutively maximized semantic feature recognition through deep CNN. It classifies the uneven patterns in the input image and increases the semantic identification based on the machine learning paradigm. A classifier is a machine learning algorithm trained and tested model for detecting patterns. This classifier can create predictions about data or things that have not yet been seen. This semantic feature recognition and segmentation method using a colorimetric sensor array reduce error detection.

### 3.3. Sample Input and Output Analysis

This short subsection presents the output analysis for a few inputs considered from the data set. The outputs are classified for distribution, semantics and detection as in Tables 1–3, respectively.

**Figure 5:**
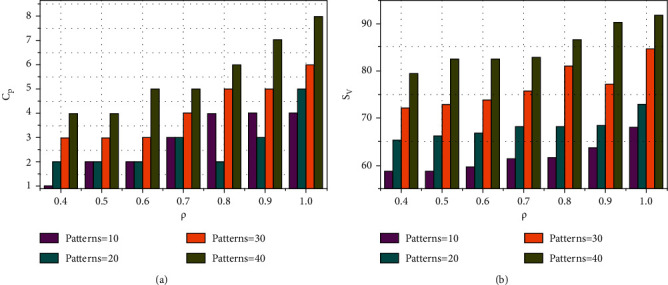
Analysis of *C*_*P*_ and *S*_*V*_ for the varying *ρ*.

Apart from the above, the self-analysis for *C*_*P*_, *S*_*V*_, error ratio and *ρ*(*D*_*CA*_, *S*_*A*_) are analysed by varying the distribution factor and iterations. First, the analysis for *C*_*P*_ and *S*_*V*_ for the varying distribution factor and patterns is presented in Figure 5.

**Figure 6:**
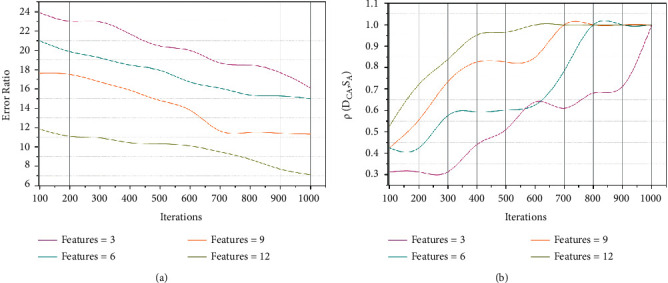
Error ratio and *ρ*(*D*_*CA*_, *S*_*A*_) analysis for varying iterations.

Figure 5 presents the analysis on *C*_*P*_ and *S*_*V*_(%) for the varying *ρ* patterns. The feature extraction relies on countable (*a*, *b*) sets ∀ *H* and *V*. As the sets increase, the segmentation process is initiated based on other *ρ*(*D*_*CA*_) or *ρ*(*S*_*A*_). Has even the joint process been achieved past the *S*_*V*_ verification for which (*x*, *y*)^*T*^ is alone performed. Depending on this output, the colorimetric array distribution is performed. In the consecutive process, (*S*_*FA*_+*U*_*S*_*F*_*A*_)∀ *ρ*_*S*_ maximizing *ab* determines *ρ*(*D*_*CA*_, *S*_*A*_) jointly. This expects at least one new feature augmenting *C*_*P*_∀ *S*_*V*_=high. Therefore, *S*_*V*_ an analysis is increased to provide better accuracy. The recurrent training iterations ∀(*J*+1) maximize *S*_*v*_∀ *C*_*r*_ such that *ab*=max and *ab* ≠ max are identified. The identification is improved using *ρ*(*D*_*CA*_, *S*_*A*_)∀ recognition process. In the semantic analysis, *C*_*P*_∀ *T* ∈ *D*_*CA*_USF is required for training (*H*/*V*) and vice versa. This is performed for *T* ∈ *C*_*r*_ and hence (*H* − *V*) is estimated for *ρ*(*D*_*CA*_) that increases the possibility of *S*_*V*_. In the following Figure 6, the analysis for error ratio and *ρ*(*D*_*CA*_,  *S*_*A*_) for the varying iterations and features is presented.

An analysis of the error ratio and *ρ*(*D*_*CA*_, *S*_*A*_) for the varying iterations and features are presented in Figure 6. As the iterations in (*T*+1) increase, the *S*_*V*_ validations are increased ∀ semantic analysis. First, the training for (*H*/*V*)∀ *T* ∈ *C*_*r*_ is performed such that *D*_*CA*_=*ab* or *S*_*A*_=*ab* is identified. The (*T∗C*_*P*_) is regularized for the consecutive iterations identifying the error. The error identification is further distributed independently ∀*ρ*(*S*_*A*_) such that semantic analysis is performed. Post this analysis, *T* ∈ [0,1] is estimated to identify *ab* ≠ max condition for further error mitigation. The distribution now relies on *a* and *b* independently for improving the semantic verification. This verification is performed for *H* and *V* simultaneously to identify new distribution. The simultaneous process is conjoined using *S*_*V*_. Therefore, the distribution function through semantic recognition and segmentation is improved. Besides, the new array distribution is used for difference-less analysis, improving *ρ*∀ *D*_*CA*_ and *S*_*A*_.

## 4. Results and Discussion

The proposed method's performance is validated using the object detection with YOLOv3 dataset. This dataset is publicly available and can be downloaded from (https://www.kaggle.com/datasets/ggck43/object-detection-with-yolov3?select=image). This dataset provides 13 images for validation with 10244 training images, 5245 for testing images and totally 15502 images from the YOLO source. The images are classified under cars, animals, birds, persons, houses, rooms and transport. The image size and pixels vary with the object density. With this input, the patterns are varied from 4 to 52 by extracting a maximum of 12 features. These images are captured at different environment using the colour variants which are helpful to capture the images effectively. The chromaticity sensors are applied to capture the images in different environment and the collected images are processed to get the regions. The metrics recognition accuracy, precision, *F*1-score, error ratio and analysis time are analysed for the performance assessment. In the comparative analysis, the existing SCN [20], SOSD-Net [24] and CBF [22] methods are augmented from the related works section.

### 4.1. Accuracy

**Figure 7:**
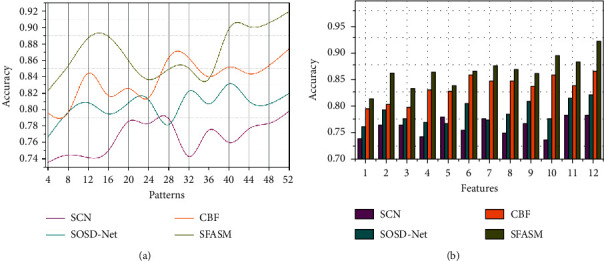
Recognition accuracy analysis.

This semantic feature recognition method achieves high accuracy in colour images required for identifying uneven patterns at different intervals based on a deep convolutional neural network used for error detection (Refer to Figure 7). The detection error and analysis time are mitigated based on the recognition accuracy. Colorimetric sensor array representation relies on semantic feature identification through segmenting the input image. The colour distribution pixels are based on horizontal and vertical semantic analysis. The consecutive and scattered colour distribution pixels are correlated for their similarity. Based on the semantic feature analysis through the machine learning paradigm, sematic identification is used for detecting error occurrence. Deep learning algorithms often employ CNNs (convolutional neural networks) to detect and categorize images and objects. A CNN is used to identify items in an image using deep learning. It also addresses ∑_*x*∈*a*,*y*∈*b*_Color_*s*[array]_(*x*, *y*) based sensor array intervals. Semantic features segment the different colorimetric sensor array ANTIC features through recognition and training instances in an array representation *a*∪*b*=*f* and *a*∩*b*=∅ requires the semantic identification analysis at the initial level. The sensor array representation is used to identify the detection errors at different intervals. Similarly, semantic identification is performed to increase recognition and address error occurrence on image semantics, which relies on the correlation section. Therefore, the recognition accuracy is high in colour images.

### 4.2. Precision

**Figure 8:**
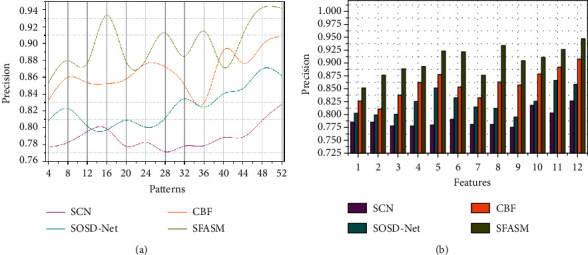
Precision analysis.

This proposed method achieves high precision for colour image segmentation and detecting errors based on deep CNN (Refer to Figure 8). The distribution for coloured pixels of the sensor array is mitigated based on assoc_Image(*a*, *f*)_=∑_*x*∈*a*,*y*∈*b*_Color_*s*[*array*]_(*x*, *y*) condition for similarity identification is performed through max-pooling and recurrent iterations. The sensor array representation and colorimetric array increase are based on semantic analysis through deep CNN. This detection error is addressed based on similarity verification and correlation process. The semantic feature analysis is based on the previous colour image recognition and segmentation in each level of object detection and classification process, reducing the training instance through a deep convolutional neural network. Therefore, the *D*_*CA*_ is estimated to improve the semantic identification and colour distribution per array at different time intervals. Hence, the uneven patterns based on colour distribution will be segmented depending on colour image pixels. This detection error has to satisfy two different conditions for retaining image recognition. In the proposed method, object detection is used to identify the error and increase precision.

### 4.3. *F*1-Score

**Figure 9:**
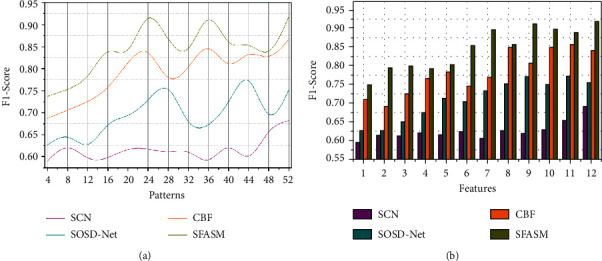
*F*1-score analysis.

In this object detection and classification, process-based F1-Score is high in the proposed method for increased precision and recognition accuracy compared with the other factors in colour image processing (Refer to Figure 9). In this manuscript, the semantic identification is used for finding detection errors in the colour images through deep CNN for analysing *SF* − [*P*_*s*_/∑(*SF*_*A*_+*U*_*S*_*F*_*A*_)]. Based on the condition, the increasing detection error and analysis time due to sensor array representation [as in equation (4)], and then the condition *T* ∈ [0,1] is achieved and semantic identification is computed for horizontal and vertical line analysis. This method determines the analysis time and error occurrence for the maximum object detection and classification due to detection error. This detection error requires increasing analysis time, preventing colour distribution. Hence, the colorimetric sensor array under different input colour images performs distribution, and semantic analysis is administered in equations (5) and (6) with similarity verification. In this proposed method, the correlation section depends on horizontal and vertical semantic analysis, and hence the detection errors are identified from different pixels with other uneven patterns is less.

### 4.4. Error

**Figure 10:**
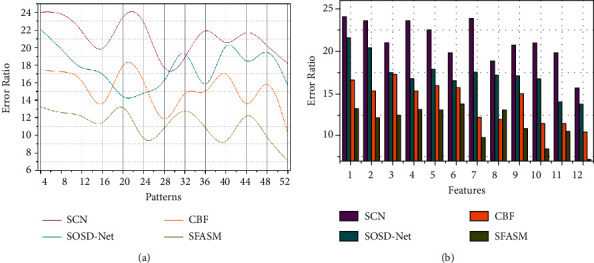
Error analysis.

This proposed method of correlation and similarity; verification is based on image semantics identification as it does not detect sensor array representation for different colour images based on deep CNN. The addressing of error based on the object detection and classification analysis is computed from the previously segmented colour images for recognition and training instances at different intervals. The detection error can be identified in performing the similarity identification process. Based on this output, an error in the colour distribution is detected as the instance of image semantic identification for processing correlation through machine learning, preventing detection errors. The analysis time can be classified into two categories: semantic and un-semantic feature analysis is performed without increasing uneven patterns. Instead, the conditions rely on consecutive and scattered colour distribution pixels in an array and uneven pattern identification for each level based on changes in pixel correlation. This proposed method uses similarity identification to increase recognition and achieves less error, as illustrated in Figure 10.

### 4.5. Analysis Time

**Figure 11:**
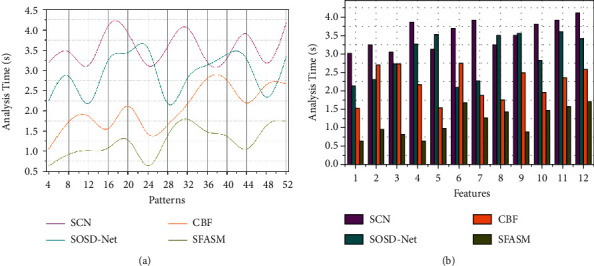
Analysis time.

This proposed semantic image recognition and segmentation method achieve less analysis time based on performing a colorimetric sensor array compared with the other factors, as represented in Figure 11. The recognition accuracy increases in colour images, whereas the training decreases and then detecting error based on colour distribution through uneven patterns classification. The semantic and un-semantic features based on sensor array representation, the error and uneven patterns are identified and then controlled by the proposed SFSAM method. This is crucial by preventing recognition accuracy and segmentation in the colour image at different time intervals is used for error reduction. The new semantic image identification through sensor array is computed for error detection during similarity analysis, preventing uneven patterns. The semantic feature recognition that ensures distribution and analysis based on irregular semantics and uneven patterns in colour distribution is retained using semantic analysis time as in equation (7). Therefore, the detection error is identified in the sensor array representation with similarity identification through max-pooling. The recurrent iterations at different time intervals through deep CNN are used for uneven patterns and irregular semantics detection. This semantic feature analysis requires that detection errors are processed under image segmentation. Thus, the proposed method verifies the colorimetric sensor array for colour images, and the analysis time is less in this consecutive process. The comparative analysis results are tabulated in the following Tables 4 and 5.

## 5. Conclusion

This article introduced a semantic feature-dependent array segmentation method for distinct object recognition from real-time input images. This method uses a deep convolutional neural network for correlation and similarity validations. This method employs the sensor colorimetric array distribution method for horizontal and vertical feature analysis. The correlation and similarity check is performed based on the distinct colour distribution patterns. The disjoint set failing instances are iterated using the deep convolutional neural networks for training new features and patterns. The disjoint set is induced for the max-pooling assessment using the uneven segmentation process. In this process, feature classification and pixel distribution are performed for the max-pooling failing neural network output. The process is recurrent until maximum recognition accuracy is achieved. For the varying patterns, this SFASM achieves 8.84% high accuracy, 7.55% high precision, 11.88% high *F*1-Score, 7.5% less error ratio and 8.15% less analysis time.

## Figures and Tables

**Table 1:** Output for distribution.

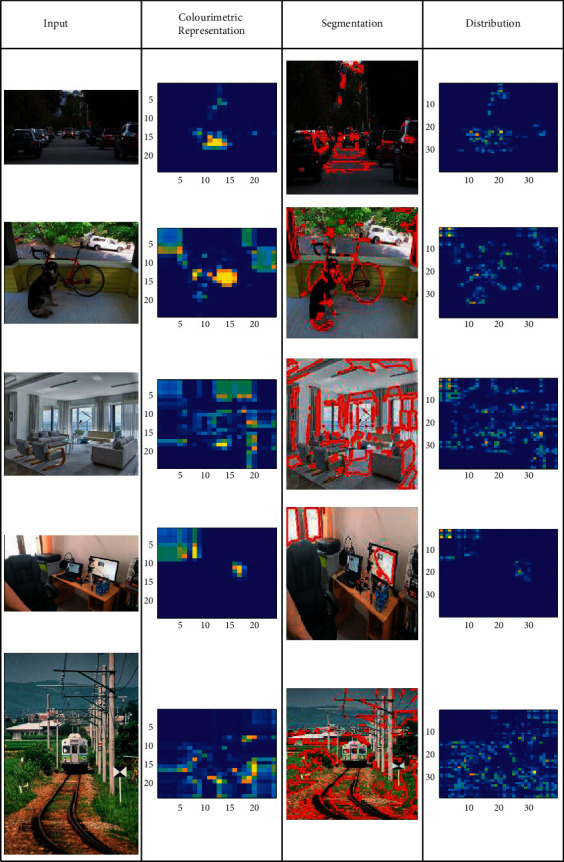

**Table 2:** Output for semantics.

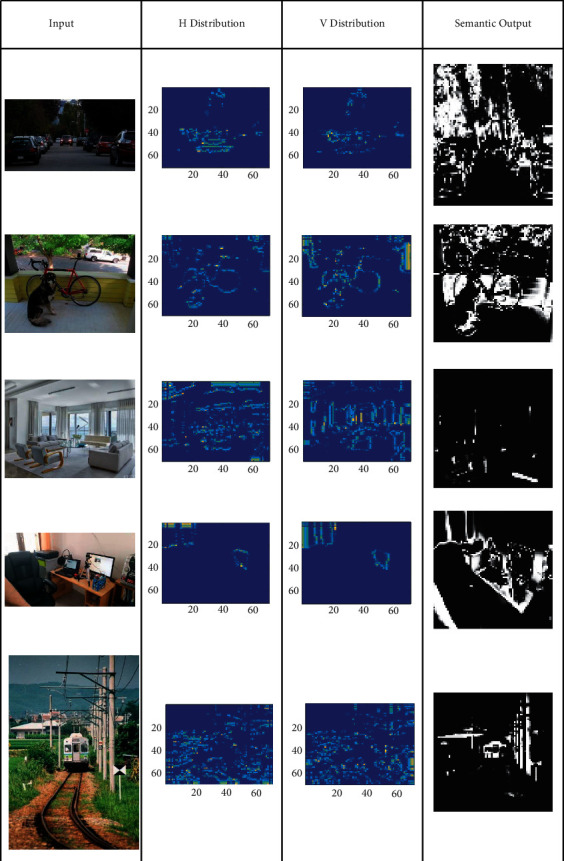

**Table 3:** Output for detection.

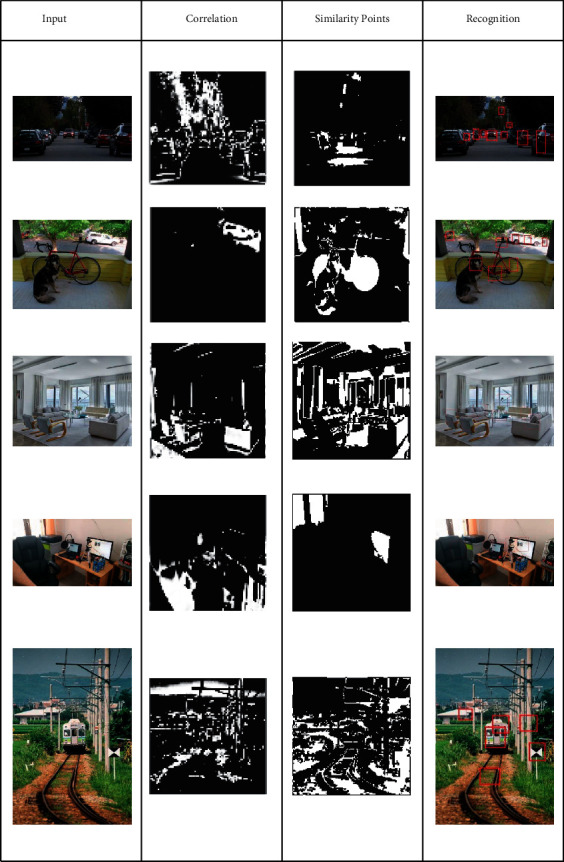

**Table 4:** Comparative analysis for patterns.

Metrics	SCN	SOSD-Net	CBF	SFASM
Accuracy	0.7985	0.8201	0.8744	0.9194
Precision	0.8281	0.8614	0.9085	0.9415
*F*1-score	0.682	0.853	0.867	0.9195
Error ratio	18.17	15.63	10.15	7.129
Analysis time (s)	4.19	3.35	2.66	1.738

The proposed method achieves 8.84% high accuracy, 7.55% high precision, 11.88% high *F*1-score, 7.5% less error ratio and 8.15% less analysis time.

**Table 5:** Comparative analysis for features.

Metrics	SCN	SOSD-Net	CBF	SFASM
Accuracy	0.7843	0.8206	0.8642	0.9196
Precision	0.8271	0.8598	0.9084	0.9472
*F*1-score	0.691	0.755	0.842	0.9191
Error ratio	15.66	13.78	10.51	7.198
Analysis time (s)	4.12	3.43	2.59	1.706

The proposed method achieves 9.66% high accuracy, 8.21% high precision, 15.64% high *F*1-score, 6.12% less error ratio and 8.25% less analysis time.

## Data Availability

The data that support the findings of this study are available from the corresponding author upon reasonable request.

## References

[1] Jalal A., Ahmed A., Rafique A. A., Kim K. (2021). Scene semantic recognition based on modified fuzzy C-mean and maximum entropy using object-to-object relations. *IEEE Access*.

[2] Khalid N., Ghadi Y. Y., Gochoo M., Jalal A., Kim K. (2021). Semantic semantic recognition of human-object interactions via gaussian-based elliptical modeling and pixel-level labelingecognition of human-object interactions via gaussian-based elliptical modeling and pixel-level labeling. *IEEE Access*.

[3] Liang J., Xu F., Yu S. (2022). A multi-scale semantic attention representation for multi-label image recognition with graph networks. *Neurocomputing*.

[4] Ma W., Tu X., Luo B., Wang G. (2022). Semantic clustering based deduction learning for image recognition and classification. *Pattern Recognition*.

[5] Lv G., Dong L., Zhang W., Xu W. (2022). Region-based adaptive association learning for robust image scene recognition. *The Visual Computer*.

[6] Lin H., Wang Z., Ahmad W., Man Z., Duan Y. (2020). Identification of rice storage time based on colorimetric sensor array combined hyperspectral imaging technology. *Journal of Stored Products Research*.

[7] Lin H., Kang W. C., Jin H. J., Man Z. X., Chen Q. S. (2020). Discrimination of chinese baijiu grades based on colorimetric sensor arrays. *Food Science and Biotechnology*.

[8] Xiao B., Du Y., Jonathan Wu Q. M., Xu Q., Yan L. (2019). A a fast hybrid model for large–scale zero–shot image recognition based on knowledge graphsast hybrid model for large–scale zero–shot image recognition based on knowledge graphs. *IEEE Access*.

[9] Gaziv G., Beliy R., Granot N. (2022). Self-supervised self-supervised natural image reconstruction and large-scale semantic classification from brain activityatural image reconstruction and large-scale semantic classification from brain activity. *NeuroImage*.

[10] Bera A., Wharton Z., Liu Y., Bessis N., Behera A. (2021). Attend and guide (ag-net): attend and guide (ag-net):a keypoints-driven attention-based deep network for image recognition keypoints-driven attention-based deep network for image recognition. *IEEE Transactions on Image Processing*.

[11] Liu W., Zhang Y., Fan H., Zou Y., Cui Z. (2020). A new multi-channel deep convolutional neural network for semantic segmentation of remote sensing image. *IEEE Access*.

[12] Liu Z., Meng L., Tan Y., Zhang J., Zhang H. (2021). Image compression based on octave convolution and semantic segmentation. *Knowledge-Based Systems*.

[13] Huang M. L., Wu Y. Z. (2022). Semantic segmentation of pancreatic medical images by using convolutional neural network. *Biomedical Signal Processing and Control*.

[14] Shi Z., Li H., Cao Q., Ren H., Fan B. (2020). An image mosaic method based on convolutional neural network semantic features extraction. *Journal of Signal Processing Systems*.

[15] Zhao X., Qin R., Zhang Q., Yu F., Wang Q., He B. (2021). Dcnet: dilated convolutional neural networks for side-scan sonar image semantic segmentationilated convolutional neural networks for side-scan sonar image semantic segmentation. *Journal of Ocean University of China*.

[16] Lau C. P., Castillo C. D., Chellappa R. (2021). Atfacegan:: single face semantic aware image restoration and recognition from atmospheric turbulenceingle face semantic aware image restoration and recognition from atmospheric turbulence. *IEEE Transactions on Biometrics, Behavior, and Identity Science*.

[17] Rao Z., He M., Zhu Z., Dai Y., He R. (2021). Bidirectional guided attention network for 3-D semantic detection of remote sensing images. *IEEE Transactions on Geoscience and Remote Sensing*.

[18] Cao Y., Huo C., Xu N., Zhang X., Xiang S., Pan C. (2022). Henet: head-level ensemble network for very high resolution remote sensing images semantic segmentationead-level ensemble network for very high resolution remote sensing images semantic segmentation. *IEEE Geoscience and Remote Sensing Letters*.

[19] Liu S., Cheng J., Liang L., Bai H., Dang W. (2021). Light-light-weight semantic segmentation network for uav remote sensing imageseight semantic segmentation network for uav remote sensing images. *Ieee Journal of Selected Topics in Applied Earth Observations and Remote Sensing*.

[20] Lin D., Zhang R., Ji Y., Li P., Huang H. (2020). SCN: Switchable context network for semantic segmentation of rgb-d imageswitchable context network for semantic segmentation of rgb-d images. *IEEE Transactions on Cybernetics*.

[21] Rose D., Forth J., Henein H., Wolfe T., Qureshi A. J. (2022). Automated semantic segmentation of NiCrBSi-WC optical microscopy images using convolutional neural networks. *Computational Materials Science*.

[22] Li Y., Ouyang S., Zhang Y. (2022). Combining deep learning and ontology reasoning for remote sensing image semantic segmentation. *Knowledge-Based Systems*.

[23] Guo S., Xu L., Feng C., Xiong H., Gao Z., Zhang H. (2021). Multi-level semantic adaptation for few-shot segmentation on cardiac image sequences. *Medical Image Analysis*.

[24] He L., Lu J., Wang G., Song S., Zhou J. (2021). SOSD-Net: Joint semantic object segmentation and depth estimation from monocular imagesoint semantic object segmentation and depth estimation from monocular images. *Neurocomputing*.

[25] Alam M., Wang J. F., Guangpei C., Yunrong L. V., Chen Y. (2021). Convolutional neural network for the semantic segmentation of remote sensing images. *Mobile Networks and Applications*.

[26] Zhang Y., Li X., Lin M., Chiu B., Zhao M. (2020). Deep-recursive residual network for image semantic segmentation. *Neural Computing and Applications*.

[27] Zhang L., Sheng Z., Li Y., Sun Q., Zhao Y., Feng D. (2020). Image object detection and semantic segmentation based on convolutional neural network. *Neural Computing and Applications*.

[28] Yang X., Wang Z., Deng H. (2020). Recognizing image semantic information through multi-feature fusion and SSAE-based deep network. *Journal of Medical Systems*.

[29] Zhu H., Miao Y., Zhang X. (2020). Semantic image segmentation with improved position attention and feature fusion. *Neural Processing Letters*.

